# Burden of disease in pediatric patients with hypophosphatasia: results from the HPP Impact Patient Survey and the HPP Outcomes Study Telephone interview

**DOI:** 10.1186/s13023-019-1167-5

**Published:** 2019-08-16

**Authors:** Eric T. Rush, Scott Moseley, Anna Petryk

**Affiliations:** 1 Children’s Mercy Hospital, University of Missouri – Kansas City School of Medicine, Kansas City, MO USA; 2 Alexion Pharmaceuticals, Inc., New Haven, CT USA

**Keywords:** Hypophosphatasia, Symptoms, Health-related quality of life, Disease burden, Survey

## Abstract

**Background:**

Hypophosphatasia (HPP) is a rare, inherited, metabolic bone disease caused by deficient tissue-non-specific isoenzyme of alkaline phosphatase activity that manifests as a broad range of signs/symptoms, including bone mineralization defects and systemic complications. The burden of disease is poorly characterized, particularly in children. This study aimed to characterize the patient-reported burden of disease among children with HPP using two survey instruments: the HPP Impact Patient Survey (HIPS) and the HPP Outcomes Study Telephone interview (HOST).

**Methods:**

Between September 2009 and June 2011, pediatric patients (aged younger than 18 years) with HPP were recruited to participate in the study via patient advocacy groups or their medical provider. Survey questions were used to capture information on patient demographics, HPP-related medical history, mobility, and health-related quality of life (HRQoL; using the 10-item Short-Form Health Survey for Children [SF-10], HIPS only).

**Results:**

Common clinical features of the 59 pediatric survey respondents (mean [standard deviation] age: 7.6 [5.1] years; 51% male) included pain (86% of patients), muscle weakness (71%), difficulty gaining weight (64%), and delayed walking (59%). Fracture was reported by 36% of patients; multiple fractures were also reported (15% of patients). Use of assistive devices for mobility was frequent among the study population (51%). In response to the SF-10, patients reported a substantial impact of HPP on their HRQoL; physical function was the most severely impaired component relative to normative data. Of patients responding to the HOST, two-thirds experienced worsening of at least one of their HPP-related signs/symptoms over a 5-year period.

**Conclusions:**

In pediatric patients, HPP is associated with a high burden of disease and a substantial negative impact on HRQoL. The burden of HPP may increase and HRQoL reduce further over time as signs/symptoms that affect HRQoL worsen or new signs/symptoms manifest.

**Electronic supplementary material:**

The online version of this article (10.1186/s13023-019-1167-5) contains supplementary material, which is available to authorized users.

## Background

Hypophosphatasia (HPP) is a rare, inherited, metabolic bone disease caused by deficiency of the tissue non-specific isoenzyme of alkaline phosphatase (TNSALP) [1]. Low TNSALP activity leads to the extracellular accumulation of its substrates including one of the key substrates involved in the pathogenesis of HPP, inorganic pyrophosphate (PPi). PPi is a potent inhibitor of bone mineralization [2]; in HPP, excess PPi contributes to skeletal hypomineralization, which can result in the premature loss of deciduous teeth, HPP-related rickets in infants and children, and osteomalacia in patients of any age [3].

HPP-related manifestations vary considerably between individuals and, aside from skeletal and dental manifestations, can include systemic complications such as respiratory difficulties, vitamin B6-dependent seizures, muscle weakness, nephrocalcinosis, and chronic pain [4–6]. Mortality is high among those who present with HPP in the perinatal and infantile periods [1]; however, it is now recognized that significant complications associated with HPP can occur at any age, and that those individuals who survive infancy or who present with HPP-related manifestations after infancy often experience a high disease burden [7]. These manifestations of HPP are often debilitating and frequently include poor growth [7–10], delayed motor milestones [9, 11], muscle weakness [7, 10], gait abnormalities, chronic bone, muscle and/or joint pain [12, 13], dental abnormalities [14], and recurrent low trauma fractures [7, 15]. As the disease progresses, these manifestations often lead to ambulatory difficulties and limitations in activities of daily living, which may result in individuals requiring assistance with daily tasks. Consequently, individuals with HPP frequently experience a diminished health-related quality of life (HRQoL) [7].

Several observational studies of pediatric patients with HPP have reported the clinical burden of the disease along specific parameters [1, 6, 10]. The results of these studies suggest that patients often experience a high burden of disease. Moreover, there are publications based on data from case reports reporting the loss of mobility over time in adults with HPP, suggesting that many patients experience a progressive worsening of the disease [16–18]. However, limited evidence on the patient-reported burden of HPP and its impact on an individual’s HRQoL exists in the literature, and to the best of our knowledge, research published to date has focused on patient-reported disease burden in adults with HPP, but not children with the disease.

The aim of the current study was to evaluate and characterize the patient-reported burden of disease in pediatric patients with HPP.

## Methods

This was a cross-sectional, survey-based study of adult and pediatric patients with HPP. Details of the materials and methods as well as the results for the adult population have been published previously [7]. This paper describes the results for the pediatric population (those younger than 18 years of age at the time the survey was administered). This paper was prepared according to the STrengthening the Reporting of OBservational studies in Epidemiology (STROBE) reporting guidelines for observational studies (Additional file 1) [19].

### Survey instruments

Two complementary survey instruments, developed by the study sponsor (Alexion Pharmaceuticals, Inc., Boston, MA, USA), were used to capture the patient-reported burden of HPP: the HPP Impact Patient Survey (HIPS) and the HPP Outcomes Study Telephone interview (HOST) [7]. The HIPS survey is presented in Additional file 2; the HOST survey has been published previously [7]. The survey questions included in the HIPS and HOST capture patient demographics, HPP-related medical history and symptoms, mobility, and HRQoL. In addition, questions included in the HOST capture change in symptoms over time. HRQoL was assessed using the validated 10-item Short-Form Health Survey for Children (SF-10), which was included as part of the HIPS (Additional file 2, questions 1–7). For questions relating to the timing of signs/symptoms, both survey instruments captured data for the following age groups: age 0 to less than 10 years, age 10 to less than 18 years, and age 18 years or older, while the HOST also captured data for age 0 to less than 1 year).

### Participants and survey administration

The HIPS was a web-based survey completed by the patients or their caregivers between September 9, 2009 and June 15, 2011. The HOST was conducted predominantly via telephone by six licensed physical therapists between December 10, 2010 and March 12, 2011. There was no specific rationale for surveying patients with the HIPS or HOST. For both surveys, patients with HPP were invited to participate via outreach from patient advocacy groups. For the HOST, some patients were also invited by their medical provider. All patients or their primary caregivers provided informed consent prior to their participation in the study. Participation did not require third-party confirmation of HPP diagnosis. All survey responses were anonymous.

### Survey data analysis

Data from both surveys were combined for analysis where possible. Data from questions unique to only one of the survey instruments were analyzed separately. For the HOST, participants who did not respond to a specific question were censored and omitted from the analyses for that question. For data from the HIPS, if it was not possible to differentiate a response of “no” from a non-response, the response was conservatively classified as “no”.

Descriptive statistics were generated for all survey participants; in all cases, the denominator was the number of responders for a given question. The difference between reported age at first manifestation of HPP and age at the time the survey instruments were administered was used to determine the length of time the patient had been living with symptoms of HPP. Means are reported as mean (standard deviation [SD]).

#### SF-10 scoring

Mean Physical Component Summary (PCS) and Mental Component Summary (MCS) scores were calculated from participant responses to the SF-10 included in the HIPS. The scores were compared with the general child population norm (mean: 50, SD: 10), which was based on a US general population sample from 1998 [20]. Means and 95% confidence intervals (CIs) were calculated for summary and individual domain scores.

## Results

### Study population

The HIPS and HOST were completed by a total of 184 respondents from 16 countries. Of these, 125 respondents were adults at the time of their participation in the survey; data from these individuals have been published [7]. The remaining 59 patients were less than 18 years of age at the time of the survey and constitute the study population (HIPS, *n* = 44; HOST, *n* = 15).

Of the pediatric survey respondents, 23.7% were patients who self-completed the survey, and 74.6% responded via their caregiver. One respondent (1.7%) completed the survey on behalf of a patient but did not identify themselves as either a caregiver or family member. Patient demographics and characteristics of the respondents are presented in Table 1. At the time of the survey, the mean (SD) patient age was 7.6 (5.1) years, and the mean (SD) reported age at onset of first HPP signs/symptoms was 0.8 (0.9) years. Both sexes were represented equally among the respondents (male, 51%; female, 49%). The mean (SD) time from the first HPP sign/symptom to participation in the study was 7.0 (5.0) years, and the mean (SD) time from the first HPP sign/symptom to diagnosis (captured in the HOST only) was 1.3 (2.3) years.

**Table 1:** Patient demographics and characteristics of survey respondents

Characteristic	Patients (*n* = 59)
Sex, n (%)	
Male	30 (50.8)
Female	29 (49.2)
Mean height, cm (SD)	
Male (*n* = 26)	106.5 (29.4)
Female (*n* = 27)	105.7 (35.1)
Mean height Z-score, (SD)^a^	
Male (*n* = 30)	−3.0 (3.5)
Female (*n* = 29)	−3.2 (2.3)
Age at survey, years	
Mean (SD)	7.6 (5.1)
Range	0–17
Age at HPP symptom onset, years	
Mean (SD)	0.8 (0.9)
Range	0–4
Duration of symptomatic disease, years	
Mean (SD)	7.0 (5.0)
Range	0–16

^a^ Mean height Z-scores were calculated using the Centers for Disease Control and Prevention method; because the date of birth or exact age in months for each patient was unknown, reported ages in whole numbers were rounded to 0.5 years (e.g. ages of 2 to < 3 years were rounded to 2.5 years)

*HPP* hypophosphatasia, *SD* standard deviation

#### Occurrence of HPP-related signs/symptoms

Participants who responded to the HOST were asked to identify their first HPP signs/symptoms (Table 2), as well as those that manifested later that were present at the time of the survey. Signs/symptoms that manifested after the respondents’ first HPP signs/symptoms are reported here as ‘additional symptoms’. Dental problems were the most commonly reported first HPP signs/symptoms (46.7% of patients); respondents also reported first HPP signs/symptoms of impaired mobility (40.0%), skeletal abnormalities (33.3%), pain (26.7%), and other HPP signs/symptoms (53.3%; other HPP signs/symptoms included failure to thrive, vomiting, lack of appetite, seizures, bulging fontanel, difficulty in gaining weight, hypercalcemia, hypercalciuria, muscle weakness and respiratory difficulties) (Table 2). It is of interest that none of the respondents reported fracture as a first HPP sign/symptom. The following additional symptoms were reported to be present at the time of the survey: impaired mobility (46.7% of patients), skeletal abnormalities (40.0%), pain (40.0%), dental problems (20.0%), fractures (6.7%), and other HPP signs/symptoms (53.3%; other HPP signs/symptoms included recurrent respiratory tract infections, nephrocalcinosis, lack of appetite, difficulty in gaining weight, tight hamstrings/calves, short stature, growth disorders, low red blood cell count and other dental/oral problems [abocclusion, malposition of teeth, dysfunction of the tongue]).

**Table 2:** First and additional signs/symptoms experienced by pediatric patients with HPP

Sign/symptom	Patients, *n* (%) (*n* = 15)
First sign/symptom	
Dental/tooth loss	7 (46.7)
Impaired mobility	6 (40.0)
Skeletal abnormalities	5 (33.3)
Pain	4 (26.7)
Fracture	0 (0.0)
Other first sign/symptom^a^	8 (53.3)
Additional sign/symptom	
Impaired mobility	7 (46.7)
Skeletal abnormalities	6 (40.0)
Pain	6 (40.0)
Dental problems	3 (20.0)
Fractures	1 (6.7)
Other additional sign/symptom^b^	8 (53.3)

Data presented were assessed by HOST only. Respondents could report up to three first signs/symptoms and up to three additional signs/symptoms

^a^ Other first signs/symptoms included failure to thrive, vomiting, lack of appetite, seizures, bulging fontanel, difficulty in gaining weight, hypercalcemia, hypercalciuria, muscle weakness, and respiratory difficulties. ^b^Other additional signs/symptoms included recurrent respiratory tract infections, nephrocalcinosis, lack of appetite, difficulty in gaining weight, tight hamstrings/calves, short stature, growth disorders, low red blood cell count, and other dental/oral problems (abocclusion, malposition of teeth, dysfunction of the tongue)

*HOST* Hypophosphatasia Outcomes Study Telephone interview, *HPP* hypophosphatasia

### Burden of disease: general

#### Pain

HPP-related pain was frequently reported by the respondents (86.4%) (Table 3), and 71.2% of the respondents reported that they had recently experienced pain (within 2–4 weeks). Of those who completed the HIPS, more than half (52.3%) reported experiencing bone pain, 43.2% reported bone pain severe enough to limit their activities, and 36.4% required medication for bone pain.

**Table 3:** History of manifestations of HPP in pediatric patients

Manifestation	Patients, *n* (%)
Pain (any HPP-related pain)	***51/ 59 (86.4)***
Joint pain^a^	27/44 (61.4)
Bone pain^a^	23/44 (52.3)
Muscle pain^a^	9/44 (20.5)
Joint and muscle manifestations	
Muscle weakness	***41/58 (70.7)***
Extremely flexible joints	21/57 (36.8)
Developmental manifestations	
Difficulty gaining weight	***38/59 (64.4)***
Delayed walking	34/58 (58.6)
Short stature	28/58 (48.3)
Seizures^a^	4/44 (9.1)
Skeletal abnormalities	
Bowing of legs or knock knees	34/59 (57.6)
Abnormally shaped chest	32/59 (54.2)
Abnormally shaped head	30/57 (52.6)
Knock knees	23/59 (39.0)
Bowing of legs	22/59 (37.3)
Bowing of arms	12/59 (20.3)
Respiratory manifestations	
Pneumonia	16/59 (27.1)
Difficulty breathing^a^	11/44 (25.0)
Fractures	
Non-vertebral fracture^a^	6/44 (13.6)
Pseudofracture^a^	3/44 (6.8)
Non-healing fracture^a^	1/44 (2.3)

Data are expressed as the number of patients who reported a particular HPP manifestation divided by the total number of patients who responded to the question. All manifestations are captured by both the HIPS and HOST unless stated otherwise. Bold italicized numbers indicate the three most frequent symptoms

^a^ Assessed by HIPS only (*n* = 44)

*HIPS* Hypophosphatasia Impact Patient Survey, *HOST* Hypophosphatasia Outcomes Study Telephone interview, *HPP* hypophosphatasia

#### Systemic manifestations of HPP

The most frequently reported systemic manifestations of HPP (excluding HPP-related pain) were muscle weakness (70.7% of patients), difficulty gaining weight (64.4%), and delayed walking (58.6%) (Table 3). The occurrence of seizure in the patients’ medical histories (assessed in the HIPS only) was the least frequently reported symptom (9.1%). Overall, 25.0% of respondents who completed the HIPS (*n* = 11/44) reported having trouble breathing.

### Burden of disease: skeletal

#### Skeletal abnormalities

The most frequently reported skeletal abnormalities were bowing of the legs or knock knees (57.6% of patients), abnormally shaped chest (54.2%), and abnormally shaped head (52.6%) (Table 3). Bowing of the arms was the least frequently reported skeletal abnormality involving long bones (20.3%).

#### Fractures

**Fig. 1:**
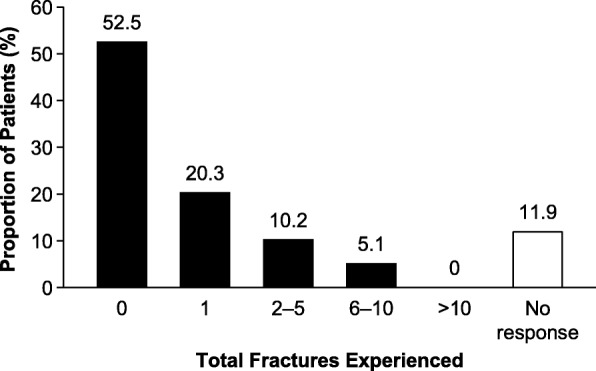
Total fractures experienced by pediatric patients with HPP (*n* = 59). There were seven respondents who did not respond to this question in the survey. HPP, hypophosphatasia

A total of 35.6% (*n* = 21/59) of patients had experienced at least one fracture (Fig. 1). Multiple fractures were also reported: 15.3% of patients reported two or more fractures, which includes 5.1% of patients who reported six or more fractures. Of respondents reporting at least one fracture, the mean (SD; range) number of fractures was 2.4 (2.2; 1–8). The types of fracture were assessed by the HIPS; non-vertebral fractures were the most commonly reported (13.6% of patients), followed by pseudofractures (6.8%) and non-healing fractures (2.3%) (Table 3).

Overall, 39.0% (*n* = 23/59) of respondents reported that their first fracture occurred from age 0 to less than 10 years, while none of the respondents reported that their first fracture occurred from age 10 to less than 18 years. The remaining participants (61%, *n* = 36/59) either did not respond to this question in the survey or had not experienced any fractures by the time of their participation in the study. A total of 13.3% (*n* = 2/15) of patients reported experiencing their first fracture from age 0 to less than 1 year (captured in the HOST only).

#### Surgeries

Patients’ histories of HPP-related surgeries were assessed in the HIPS (Table 4). More than one-third (36.4%) of respondents reported one or more HPP-related surgeries, with surgery on the skull (20.5%) being the most frequently reported. Of those patients who participated in the HOST, 35.7% (*n* = 5/14) reported having undergone surgery involving internal surgical instrumentation (screws, rods, plates, or any internal fixation).

**Table 4:** History of surgeries in pediatric patients with HPP

Type of surgery	Patients, *n* (%) (*n* = 44)
Any surgery	16 (36.4)
Surgery on the skull	9 (20.5)
Osteotomy	5 (11.4)
Root canal	4 (9.1)
Club foot surgery	3 (6.8)
Stapling of growth plates	2 (4.5)
Fracture fixation	2 (4.5)
Bone biopsy	1 (2.3)

The types of surgery presented were assessed by HIPS only

*HIPS* Hypophosphatasia Impact Patient Survey, *HPP* hypophosphatasia

### Burden of disease: function

#### Mobility

**Fig. 2:**
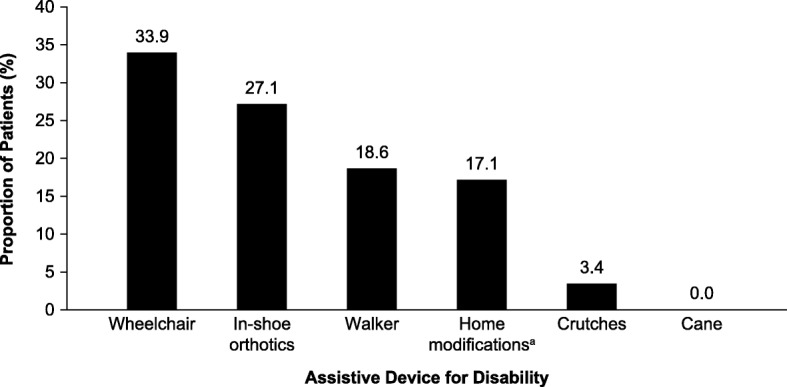
Proportion of pediatric patients with HPP using assistive devices for disability. Note that patients could report the use of more than one type of assistive device. Data presented were assessed by both HIPS and HOST unless otherwise stated (*n* = 59). ^a^Home modifications consisted of alterations to the kitchen, bathroom, bedroom, and/or entryways. These were assessed by HIPS only (*n* = 44). There were five respondents who did not respond to this question in the survey. HIPS, Hypophosphatasia Impact Patient Survey; HOST, Hypophosphatasia Outcomes Study Telephone interview; HPP, hypophosphatasia

A total of 50.8% (*n* = 30/59) of the respondents reported using an assistive device and/or braces for mobility at some time. The mean (SD; range) number of assistive devices used per patient was 1.2 (1.2; 0–11). At the time of the survey, the most frequently reported assistive devices in use were wheelchairs (33.9% of patients) and in-shoe orthotics (27.1%), while a smaller number reported use of a walker (18.6%) (Fig. 2). Of the respondents participating in the HIPS, 17.1% reported that home modifications had been made as a consequence of their HPP. These modifications consisted of alterations to the kitchen, bathroom, bedroom, and/or entryways. The HOST assessed worsening or improvement in patients’ ability to walk. Overall, 40.0% of respondents (*n* = 6/15) reported that their ability to walk had worsened since the time of their diagnosis of HPP, while 33.3% (*n* = 5/15) reported an improvement.

#### Activities of daily living

**Fig. 3:**
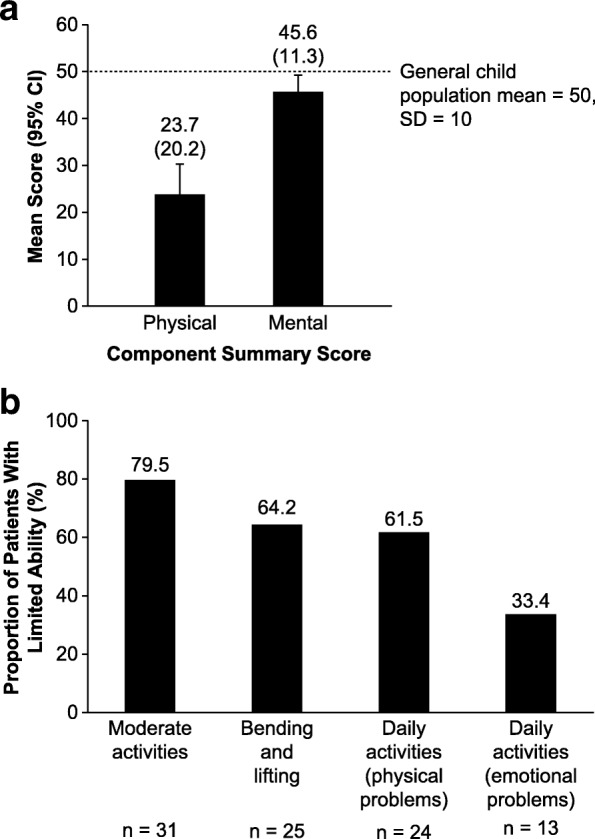
Impact of HPP on **a**) HRQoL and **b**) activities of daily living among pediatric patients. **a** Physical Component Summary and Mental Component Summary scores as assessed by the SF-10 (HIPS, *n* = 44). Mean (SD) scores are given above each bar. **b** Self-reported (or caregiver/family member-reported) inability to perform activities of daily living (HIPS, *n* = 44). Information in brackets is the reason given for the specific inability. There were five patients (or caregivers/family members) who did not respond to this part of the HIPS. HIPS, Hypophosphatasia Impact Patient Survey; HPP, hypophosphatasia; HRQoL, health-related quality of life; SD, standard deviation; SF-10, 10-item Short-Form Health Survey for Children

Most respondents reported that their health impaired their physical and mental function, as measured by the SF-10, which was administered as part of the HIPS (Fig. 3a). Mean (95% CI) PCS and MCS scores for pediatric patients with HPP were 23.7 (17.2, 30.3) and 45.6 (41.9, 49.3), respectively, both of which are lower than the general child population mean of 50 (SD: 10) [20]. In addition, between 33.4 and 79.5% of the HIPS respondents reported that they were limited in their ability to undertake moderate activities (such as standing from a sitting position and walking), bending, lifting, and daily activities (such as attending school) as a consequence of their physical and/or emotional challenges (Fig. 3b).

### Burden of disease: longitudinal evaluation

The HOST assessed whether respondents’ first HPP signs/symptoms had improved, worsened, or remained the same from the time of their first occurrence to the time of the survey. Overall, 7 respondents reported improvements in one or more of their initial signs/symptoms, while 3 reported worsening and 4 reported no change in their first signs/symptoms.

**Fig. 4:**
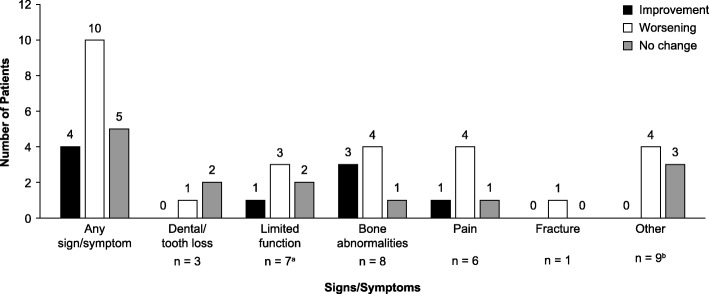
Proportion of pediatric patients with HPP experiencing an improvement, worsening, or no change in signs/symptoms compared with 5 years ago. This was assessed by HOST only (*n* = 15). Patients’ first HPP-related signs/symptoms were excluded from this analysis; respondents could report up to three signs/symptoms that manifested after their first HPP signs/symptoms. Other HPP signs/symptoms included recurrent respiratory tract infections, nephrocalcinosis, lack of appetite, difficulty in gaining weight, tight hamstrings/calves, short stature, growth disorders, low red blood cell count, and other dental/oral problems (abocclusion, malposition of teeth, dysfunction of the tongue). Data for any sign/symptom are the numbers of patients who reported an improvement, worsening, or no change in their additional signs/symptoms compared with 5 years prior to their participation in the survey in response to the free-text survey question “What additional symptoms related to HPP do you have today?”. ^a^One respondent did not indicate whether their signs/symptoms had improved or worsened, or were unchanged compared with 5 years ago. ^b^Two respondents did not indicate whether their signs/symptoms had improved, worsened, or were unchanged compared with 5 years ago. HOST, Hypophosphatasia Outcomes Study Telephone interview; HPP, hypophosphatasia

In addition, respondents who completed the HOST were asked whether their additional HPP signs/symptoms had improved, worsened, or remained the same compared with their severity 5 years prior to the survey. Improvements in one or more additional signs/symptoms were reported by 4 patients, worsening was reported by 10 patients, and 5 patients reported no change in one or more additional signs/symptoms (Fig. 4).

## Discussion

This study characterizes the impact of HPP on the HRQoL of pediatric patients with HPP as reported by the patients themselves or their caregivers. The findings of this study suggest that pediatric patients experience a high burden of disease and reduced HRQoL, and that burden of disease may increase and HRQoL reduce further over time as signs/symptoms that impact HRQoL worsen or new signs/symptoms manifest. Two-thirds of the patients reported that at least one of their HPP-related additional signs/symptoms had worsened over a 5-year period. Overall, the majority of pediatric patients with HPP reported that the disease often limited their ability to perform activities of daily living. Most patients identified physical reasons for these limitations, while 33.4% of patients surveyed stated that emotional problems were impeding their ability to perform daily activities. As such, this study highlights the substantial morbidity that children with HPP frequently experience, and that many patients experience worsening of their disease over time.

The findings of this pediatric analysis are broadly consistent with those reported previously in the analysis of 125 adults with HPP, which showed that HPP can confer a high burden of disease in adulthood [7]. Using the 12-item Short-Form Health Survey version 2, that analysis indicated that HPP has a wide-ranging and substantial impact on the HRQoL of adult patients, and that these patients often experience pain (95% of survey respondents), fractures (86%), multiple fractures (74%), muscle weakness (62%), and abnormal gait (52%). A high percentage (60%) of the patients surveyed also reported using assistive devices for mobility [7]. Similarly, in this analysis of pediatric patients, the most commonly reported signs/symptoms were pain, muscle weakness, and challenges with mobility. However, the reported frequency of occurrence of several HPP-related manifestations differed substantially between the two patient populations. For example, difficulty gaining weight, delayed walking, and skeletal abnormalities were reported more frequently by pediatric patients with HPP than by adult patients with HPP. Some of these differences may be attributed to growth during childhood, meaning some developmental and skeletal abnormalities are more likely to occur in children than in adults.

Notably, fractures occurred less frequently in pediatric patients than in adults with HPP (36% versus 86%, respectively) and none of the pediatric survey respondents reported fracture to be a first HPP sign/symptom. The prevalence of more than one fracture among children with HPP (15%) was similar to the prevalence in healthy children, which data indicate is 15–20% [21]. In general, the occurrence of two or more long bone fractures in those under 10 years of age, or three or more long bone fractures in those from age 10 to less than 16 years, or the occurrence of a vertebral compression fracture is considered abnormal [21]. While detailed information about the mechanism of fracture or fracture location was not collected as part of this study, it is remarkable that 10.2% of patients have experienced 2–5 fractures, and 5.1% of patients reported 6–10 fractures. The report of pseudofractures and non-healing fractures is also noteworthy, as they are reflective of underlying HPP-related rickets or osteomalacia [4, 18]. Pseudofractures are considered to be a type of insufficiency fracture that can occur in several forms of rickets and osteomalacia, both genetic (e.g. HPP and X-linked hypophosphatemia) and acquired (e.g. severe vitamin D deficiency), [22] and therefore the occurrence of such fractures is not expected among the general pediatric population. Non-healing fractures occur in the general pediatric population in the absence of specific risk factors at a rate of approximately 0.002% per fracture [23].

Consistent with the previous study among adults with HPP, the respondents in the current survey scored below the general child population norm in both the physical and mental components of the SF-10. Although the mean MCS scores for pediatric and adult patients reported by the two studies were comparable (45.6 versus 46.4, respectively), the mean PCS score was substantially lower among pediatric patients with HPP than among the adult patients who were surveyed (23.7 versus 32.8, respectively), and the general population (50.0). The high patient-reported physical burden of HPP is reflected in the prevalent use of assistive devices, including wheelchairs and in-shoe orthotics, by the adults and children surveyed in both analyses [7]. While overall levels of use of assistive devices were similar in adults and pediatric patients, the types of devices used differed between the two study groups. For example, only 3% of children reported using crutches compared with 30% of adults [7].

Although studies looking at the burden of disease from the patient perspective offer important insights into how patients themselves perceive their HRQoL, the data collected from such studies have several notable limitations. In this study, HPP diagnosis was not confirmed by a third party or by examination of patients’ medical records, and the criteria by which a diagnosis of HPP was made were not defined. Also, the study population may not be fully representative of the overall population of individuals with HPP because patients were invited to participate in the research through their association with patient advocacy groups or a referral by their clinician. Therefore, an element of self-selection was permitted by the study design, which may have biased the study population towards one with a disproportionate number of highly motivated patients/caregivers. In addition, the findings from this study are based on a relatively small cohort of patients but, considering the rarity of HPP, may be considered adequate for drawing conclusions based on the analyses conducted. Recall bias is an inherent limitation of patient-reported data. This study required respondents to state how their reported first signs/symptoms of HPP changed from the time of their first occurrence to the time of the survey, and to state how their reported additional HPP signs/symptoms had changed compared with 5 years before participating in the survey. These long recall periods may have reduced the accuracy of these patient-reported data.

## Conclusions

To the best of our knowledge, this is the first study to characterize the patient-reported burden of disease among children with HPP. This study highlights that HPP is associated with a high disease burden and can significantly affect patients’ HRQoL. Although some patients can experience improvement of some signs or symptoms, two-thirds experienced worsening of at least one of their HPP-related signs/symptoms over time. Future analyses of data collected by the HPP registry established in 2015 should extend our current understanding of the natural history, clinical course, and burden of disease in children and adults with HPP.

## Additional files

Additional file 1: STROBE Statement: checklist of items that should be included in reports of observational studies. STROBE, STrengthening the Reporting of OBservational studies in Epidemiology. (PDF 17 kb)

Additional file 2: Hypophosphatasia Impact Patient Survey (HIPS). (PDF 95 kb)

## Data Availability

The HIPS survey is provided in Additional file 2 (the HOST survey has been published previously [7]; however, survey responses cannot be shared owing to the fact that HPP is a rare disease, and therefore the identities of the respondents can be easily determined.

## Abbreviations

CI: Confidence interval
HIPS: Hypophosphatasia Impact Patient Survey
HOST: Hypophosphatasia Outcomes Study Telephone interview
HPP: Hypophosphatasia
HRQoL: Health-related quality of life
MCS: Mental Component Summary
PCS: Physical Component Summary
PPi: Inorganic pyrophosphate
SD: Standard deviation
SF-10: 10-item Short-Form Health Survey for Children
STROBE: STrengthening the Reporting of OBservational studies in Epidemiology
TNSALP: Tissue non-specific isoenzyme of alkaline phosphatase
