# Supplementary material for: Burden of disease in pediatric patients with hypophosphatasia: results from the HPP Impact Patient Survey and the HPP Outcomes Study Telephone interview
Source: Orphanet J Rare Dis. 2019 Aug 16;14:201. doi: 10.1186/s13023-019-1167-5 (PMC6698035; doi:10.1186/s13023-019-1167-5)
Supplement: Supplementary file 2 — Hypophosphatasia Impact Patient Survey (HIPS). (PDF 95 kb) [file 13023_2019_1167_MOESM2_ESM.pdf]

Hypophosphatasia Impact Patient Survey (HIPS) – English

Today's Date _______ /________/________
Month Day Year

Person Completing Questionnaire

_____ Patient _____ Parent/ Guardian or Caregiver of Patient _____ Other

If you are not the patient, please complete the remainder of the questionnaire by providing information about the person who has b
diagnosed with hypophosphatasia.

Gender_____ Male _____ Female
Current Age _____ years
Country of Residence:

1. **In general, would you say your child's health is:**

Excellent	Very good	Good	Fair	Poor
☐1	☐2	☐3	☐4	☐5

2. **During the past 4 weeks, has your child been limited in any of the following activities due to HEALTH problems?**

	Yes, limited a lot	Yes, limited some	Yes, limited a little	No, not limited
a. Doing things that take some energy such as riding a bike or skating?	☐1	☐2	☐3	☐4
b. Bending, lifting, or stooping?	☐1	☐2	☐3	☐4

3. **During the past 4 weeks, has your child been limited in the KIND of schoolwork or activities with friends he/she could do because of PHYSICAL health problems?**

Yes, limited a lot	Yes, limited some	Yes, limited a little	No, not limited
☐1	☐2	☐3	☐4

4. **During the past 4 weeks, has your child been limited in the KIND of schoolwork or activities with friends he/she could do because of EMOTIONAL or BEHAVIORAL problems?**

Yes, limited a lot	Yes, limited some	Yes, limited a little	No, not limited
☐1	☐2	☐3	☐4

5. **During the past 4 weeks, how much bodily pain or discomfort has your child had?**

None	Very mild	Mild	Moderate	Severe	Very severe
☐1	☐2	☐3	☐4	☐5	☐6

6. **During the past 4 weeks, how satisfied do you think your child has felt about his/her friendships?**

Very satisfied	Somewhat satisfied	Neither satisfied nor dissatisfied	Somewhat dissatisfied	Very dissatisfied
☐1	☐2	☐3	☐4	☐5

7. **During the <u>past 4 weeks</u>, how satisfied do you think your child has felt about his/her life overall?**

Very satisfied	Somewhat satisfied	Neither satisfied nor dissatisfied	Somewhat dissatisfied	Very dissatisfied
☐1	☐2	☐3	☐4	☐5

8. **During the <u>past 4 weeks</u>, how much of the time do you think your child acted bothered or upset?**

All of the time	Most of the time	Some of the time	A little of the time	None of the time
☐1	☐2	☐3	☐4	☐5

9. **Compared to other children your child's age, in general would you say his/her behavior is:**

Excellent	Very good	Good	Fair	Poor
☐1	☐2	☐3	☐4	☐5

Current Height _____ feet ____ inches OR _____ centimeters

Current Weight _____ pounds OR _____ kilograms

Do other members of your family have hypophosphatasia? _____ YES _____ NO

If yes, how many of your relatives have been diagnosed with hypophosphatasia? ______

At what age did you first experience symptoms of hypophosphatasia? _____ Years

What were the first symptoms of hypophosphatasia that you experienced?

____

____

<u>MEDICAL HISTORY</u>

Have you ever been diagnosed with or treated for any of the following conditions? (Check all that apply)

<u>Developmental</u>

_____ Difficulty gaining weight (feeding difficulties as an infant/child)
_____ Delayed walking (first walked at 15 months of age or later)
_____ Delayed talking (speech difficulties)
_____ Short stature (woman < 5 feet tall; man < 5 feet, 4 inches)
_____ Seizures

<u>Bone</u>

_____ Abnormally shaped chest (rib cage abnormalities)
_____ Abnormally shaped head (skull)
_____ Bowing of legs (rickets in legs)
_____ Bowing of arms (rickets in arms)
_____ Knock knees (knees touch but ankles do not touch when standing upright)
_____ Vertebral fracture (broken bone in back)
_____ Non-vertebral fracture (broken bone anywhere other than back)
_____ Club foot deformity
_____ Bone pain (arms, ribs, back, legs, feet) severe enough to force you to limit your activities
_____ Bone pain (arms, ribs, back, legs, feet) severe enough to require pain medication
_____ Fractures that won't heal
_____ Pseudofractures (incomplete fractures or fissures)
_____ Unusual gait or way of walking/running

<u>Joint</u>

_____ Extremely flexible joints (hypermobility)
_____ Joint swelling
_____ Joint pain (neck, shoulder, elbow, wrist, hips, knees, ankles)

If you do experience joint pain:
Is it severe enough to force you to limit your activities? _____ Yes _____ No
Is it severe enough to require pain medication? _____ Yes _____ No

Pulmonary
_____ Difficulty breathing
_____ Pneumonia

Dental/Oral
_____ Premature tooth loss (lost first baby tooth before 5 years of age)
_____ Tooth abscess
_____ Excessive cavities
_____ Loss of adult teeth
_____ Difficulty eating/swallowing

Muscle
_____ Muscle weakness
_____ Muscle pain

Renal
_____ Kidney stones
_____ Nephrocalcinosis (calcium deposits in the kidneys)

Other
_____ High calcium levels in blood
_____ High phosphate levels in blood
_____ Gout

Have you ever fractured a bone? _____ YES _____ NO

If yes, please answer the following questions.

When did your first fracture occur?
_____ Childhood
_____ Adolescence
_____ Adult Age

Approximately how many fractures have you had? _____

Please indicate the location of the fracture and the approximate date of each fracture:

Location of Fracture (Foot, Arm, Leg, Back, Ribs, etc.)	Year

How many of your fractures were caused by…? (insert number of fractures)
_____ Trauma (impact, force, accident)
_____ Other Reasons (fatigue/exhaustion fractures, no obvious reason)

How many of your fractures were… ? (insert number of fractures)
_____ Complete (impact, force, accident)
_____ Incomplete (pseudofractures)

If you have had incomplete or pseudofractures…

How long did it take for the pseudofractures to be diagnosed?
_____ Immediately
_____ 2 to 4 weeks
_____ 1 to 2 months
_____ 2 to 3 months
_____ Longer than 3 months

Approximately how long did it take for the pseudofractures to heal?
_____ 1 to 2 months
_____ 2 to 3 months
_____ 3 to 6 months
_____ 6 to 12 months
_____ More than 1 year

Have you ever had any of the following medical procedures?
_____ EMG (test that measures electrical activity in muscles)
_____ EEG (test that measures electrical activity in your brain using electrodes)
_____ Nerve conduction study (test that measures electrical activity in nerves)
_____ Muscle biopsy
_____ Kidney ultrasound
_____ Barium swallow study (x-ray of digestive tract after drinking liquid that shows up on x-ray)
_____ Bone biopsy

Have you ever had any of the following surgeries?
_____ Skull surgery
_____ Stapling of growth plates (8-plates)
_____ Fracture fixation with plates and screws
_____ Fracture fixation with steel or titanium rods within the bone
_____ Fixateur extern
_____ Joint replacement (specify joint)
_____ Rib replacement
_____ Osteotomy (surgical correction of a bone deformity that required cutting into bone)
_____ Club foot corrective surgery
_____ Dental implant surgery (to replace missing teeth)
_____ Root canal surgery

If you have had surgery for any complication of hypophosphatasia, please indicate the reason for the surgery and the approximate date of the surgery:

Reason for Surgery	Date (Year)

Are you currently receiving any of the following out-patient health services?
_____ Physical Therapy
_____ Respiratory Therapy
_____ Home Health Care
_____ Massage Therapy
_____ Acupuncture
_____ Occupational Therapy
_____ Dietary Therapy/Nutritional Consultation

MEDICATIONS

Please list any PAIN medications you are currently taking including name, dosage and frequency.

____
____
____
____

Please list any OTHER medications you are currently taking including name, dosage and frequency.
____
____
____
____
____

MOBILITY

Has your home been modified due to your disease? _____ YES _____ NO

If yes, check all areas of your home that have been modified:
_____ Kitchen _____ Thresholds/Entryways
_____ Bedroom _____ Bathroom

Please indicate if you are using paid assistance for the following activities due to hypophosphatasia:
_____ Household activities (cleaning, shopping, cooking, etc.)
_____ Family care (watching over children, making appointments, etc.)
_____ Nursing care (medications, equipment, etc.)
_____ Bodily care (bathing, grooming, etc.)

Please indicate which of the following aids you are using or have ever used. Check all that apply:

_____ mechanical lift (car)	_____ adjustable chair (not a wheelchair)
_____ handicap ramps	_____ adjustable bed
_____ handrails	_____ orthotics (braces)
_____ cane	_____ stander
_____ crutches	_____ motorized scooter
_____ walker	_____ toilet lift
_____ manual wheelchair	_____ power wheelchair
_____ shower chair	_____ none of the above

Are you currently using a wheelchair? _____ YES _____ NO
When did you start using the wheelchair? _______ /________
Month Year
Are you currently using a walking device (cane, walker, etc.) _____ YES _____ NO
When did you start using a walking device? _______ /________
Month Year

RESPIRATORY

Do you currently use a respiratory support device(s)? _____ YES _____ NO
If yes, please check all that apply:
_____ Ventilator (with trach tube) _____ CPAP _____ BiPAP _____ Supplemental oxygen
____ Other, please specify ________________________

How has your hypophosphatasia developed over the past 5 years?

_____ Improved _____ Worsened _____ Unaltered

List the three symptoms or complications from hypophosphatasia that interfere most with your life
1. ____
2. ____
3. ____

Thank you for your time and effort in completing the survey! To show our appreciation, we will donate a small grant of $20 USD or 15Euro (use symbol, French and German versions) to the patient association of your choice. Please select the patient group below:
US Soft Bones
HPP ev
Hypophosphatasie Europe
CORD

If you wish to be acknowledged for sending the donation, please provide your first and last name here:_________________ (this name will be sent only to selected patient group above, and not to the survey sponsor).
